# Successful management of ectopic kidney stones in a patient with situs inversus totalis: a rare case report

**DOI:** 10.1186/s12894-022-01137-x

**Published:** 2022-11-10

**Authors:** Marah Mansour, Abdulmonem Naksho, Yassamine Ouerdane, Tamim Alsuliman, Hani Almozawer, Khaled Alrebdawi

**Affiliations:** 1 Faculty of Medicine, Tartous University, Tartus, Syrian Arab Republic; 2 MSc Global Public Health Nutrition, School of Life Sciences, University of Westminster, London, UK; 3 Faculty of Medicine, Al-Baath University, Homs, Syrian Arab Republic; 4 Faculty of Medicine, Saad Dahlab University, Blida, Algeria; 5 Hematology and Cell Therapy Department, Saint-Antoine Hospital, AP-HP, Sorbonne University, Paris, France; 6 Department of Urological Surgery, Surgical Kidney Hospital, Damascus, Syrian Arab Republic

**Keywords:** Situs inversus, Ectopic kidney, Nephrectomy, Stones, Case report

## Abstract

**Background:**

Situs inversus totalis is a very rare congenital anatomical variation, in which all thoracic and abdominal organs are right-left inverted. This condition is associated with an increased risk of organ malformations including ectopic kidney, which is a very rare combination.

**Case presentation:**

A 56-year-old male presented with colicky left iliac pain associated with nausea, vomiting, and irritative lower urinary symptoms. The patient has a medical history of recurrent lower urinary infections and a family history of situs inversus totalis. Radiological images demonstrated dextrocardia, situs inversus totalis of all the abdominal organs, and an ectopic pelvic kidney on the left side, with 4 stones inside it. Left nephrectomy was performed due to extensive renal damage. At discharge and during follow-up, the patient's condition was satisfactory and stable.

**Conclusions:**

The ectopic kidney may present diagnostic and therapeutic challenges when associated with situs inversus.

## Background

Situs inversus totalis (SIT) is a rare congenital condition that is characterized by an anatomical variation. It is only found in 1/8000 to 1/25,000 of the general population [1]. It can also be termed “mirror man”, as all thoracic and abdominal viscera are reversed 180° (complete right-left inversion), including the heart, liver, spleen, stomach, and bowels [2]. In general, this rare anomaly is usually discovered incidentally during thoracic and abdominal imaging. Although the mechanism responsible for SIT is not fully understood yet, it is believed to result from chromosomal abnormalities that lead to a reversal of right-left polarity [1]. SIT in itself is not thought to influence overall health or life expectancy. However, patients with SIT have an increased risk of a wide range of organ abnormalities (such as cardiac, splenic, and hepatobiliary malformations) [3]. Renal anomalies, including agenesis, dysplasia, hypoplasia, ectopia, polycystic kidney, and horseshoe kidney, have also been reported to be associated with SIT Laparoscopy is considered the standard treatment for both radical and simple nephrectomy [4]. SIT with an ectopic kidney is very rarely reported in the literature.

## Case presentation

**Fig. 1:**
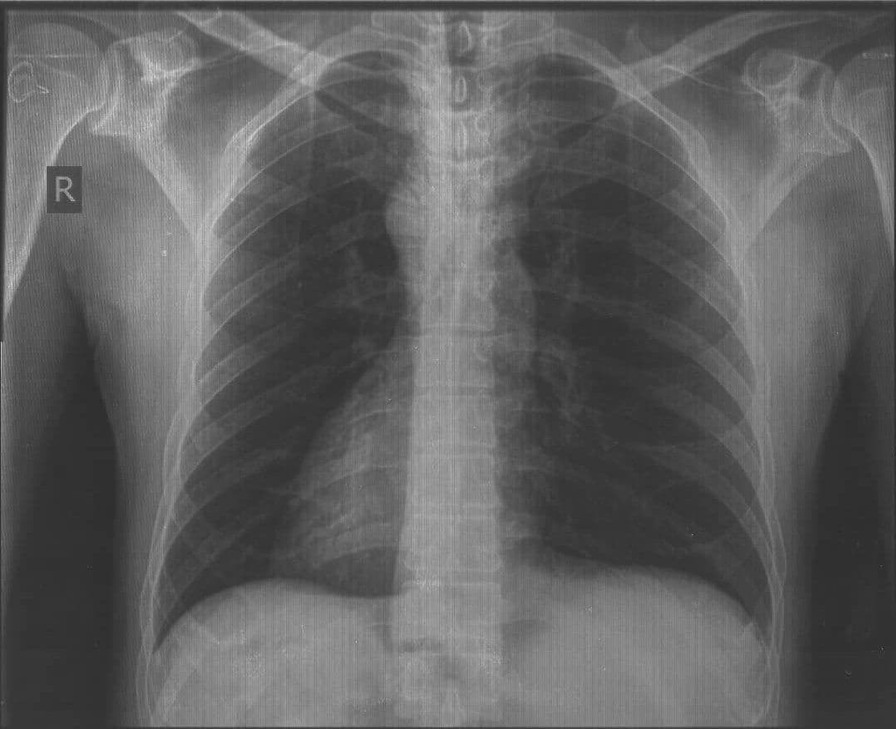
Chest X-ray showing dextrocardia

**Fig. 2:**
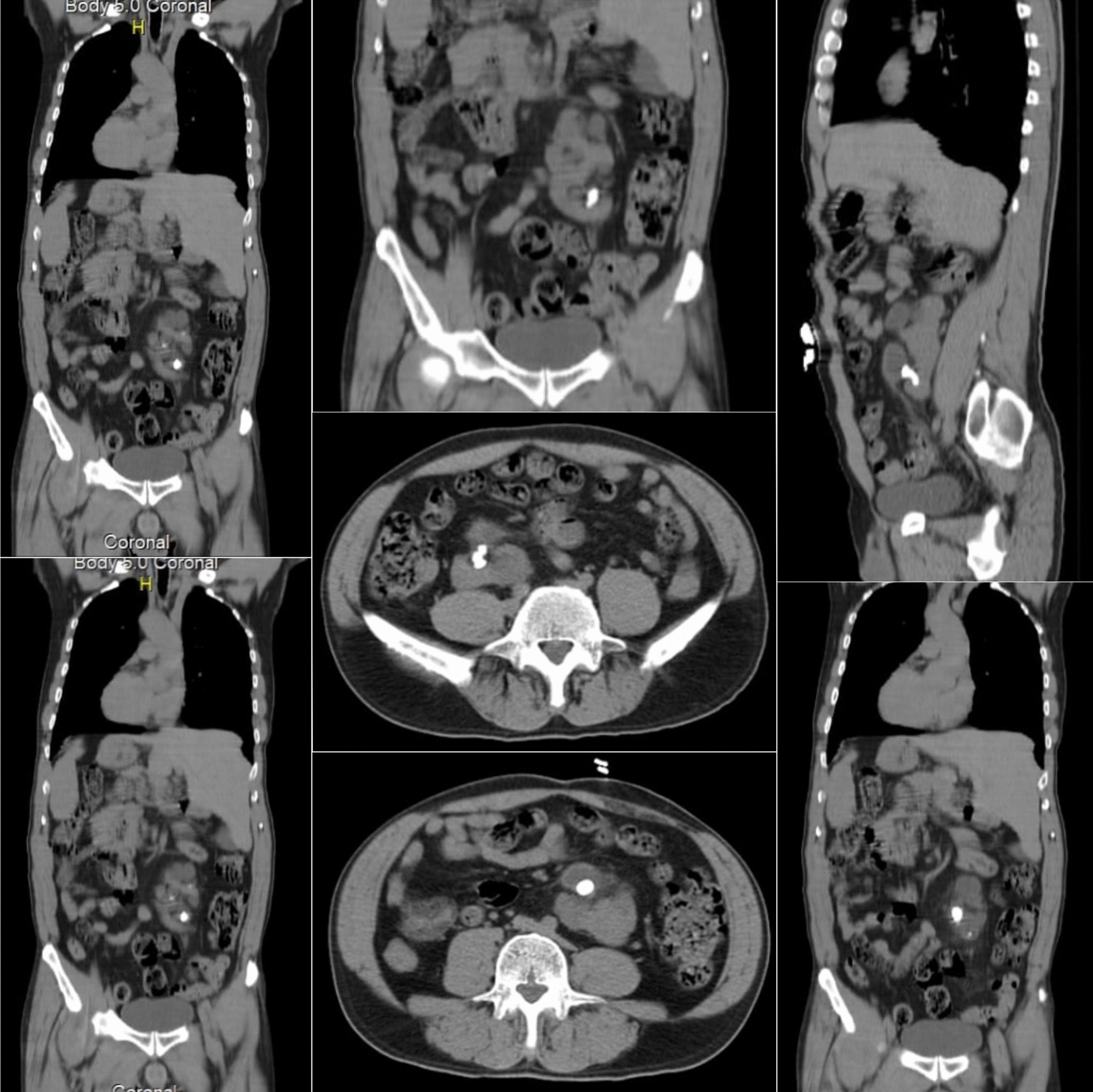
Different planes of CT KUB showing different kidney stones

A 56-year-old male was admitted to the Department of Urology Surgery with left iliac pain associated with nausea, vomiting, and irritative lower urinary symptoms. The pain was colicky in nature, responding well to analgesics, and had been occurring intermittently over the previous few years. The patient had a medical history of recurrent lower urinary infections that were diagnosed and successfully treated without the need for hospitalization. The patient’s family history is significant for SIT, with his daughter and his brother both diagnosed with this anatomical variation. The patient’s parents were not consanguineous, while the patient and his wife are consanguineous. A chest X-ray showed dextrocardia (Fig. 1). Ultrasound and computed tomography of the abdomen and pelvis demonstrated SIT of all the abdominal organs, with an ectopic pelvic kidney on the left. The left Kidney was located in the pelvis alongside the vertebrae L3–L5, with one large pelvic stone (1*2 cm) and 3 smaller stones (≤ 1 cm) in the lower pole calyx shown on CT KUB (Fig. 2). This led to a distended left renal pelvis, along with thickening (4 mm) of its wall, indicating chronic inflammation. Ultrasound of the urinary tract showed distension following an obstruction at the left ureteropelvic junction. A renal scan using Tc-99 m diethylene-triamine-pentaacetate revealed that the relative function of the left kidney is 15.5% of the total function of both kidneys, with a glomerular filtration rate of 14 ml/min. There were no associated comorbidities or other congenital anomalies. A left nephrectomy via open surgery was planned due to extensive renal damage and limited access to laparoscopic tools. During the surgery, the pelvic ectopic kidney was found to be anteriorly rotated with the anterior renal pelvis and several abnormally located blood vessels. A Gibson surgical incision was made, retroperitoneal access was obtained, left ureter and renal vessels were isolated and clamped, the left kidney was dissected and removed successfully. Left kidney’s pathological studies reported chronic interstitial nephritis and hemorrhage, along with Florid Von Brunn’s nests and squamous metaplasia in the pelvis and calyces, without evidence of neoplasm. The patient’s condition was very good on the 3-monthly follow-up visits, with significant improvement in renal function and no serious complications.

## Discussion

SIT, also termed mirror man, is a rare genetic condition in which abdominal, thoracic organs, and blood vessels are reversed 180°. The incidence rate is thought to be in the range of 1 in 8000 to 1 in 25,000 [1, 5]. SIT is frequently associated with kartagener syndrome, which describes a constellation of cardiovascular [6] and hepatobiliary abnormalities [7]. Renal anomalies, including agenesis [8], dysplasia [9], and hypoplasia [10] are the most prominent reported association with SITs. Genes might support this anomaly [11], as similar cases of SIT were reported in our patient’s family (his brother and daughter). The mechanism of this condition is not fully understood. Based on advanced molecular biology techniques, several genes were identified to be involved in this asymmetry such as nodal or lefty in mice [11], other modifier genes or environmental factors are also likely to contribute [12].

In our case, we report a rare association between SIT and an ectopic kidney with a large pelvic stone and an obstruction of the left ureteropelvic junction. This obstruction occurs in 22–37% of ectopic kidneys [13].

Treatment of ectopic kidney stones (EKS) is considered challenging for the urologist [14, 15]. According to the European guidelines of Urology (EGU), non-invasive and minimally invasive treatments such as shock wave lithotripsy (SWL) and percutaneous nephrolithotomy (PCNL) represent the first choice in the management of kidney stones. However, under aberrant circumstances such as an ectopic kidney, laparoscopic-assisted PNL represents a safe and effective treatment approach [15].

Moreover, retrograde intrarenal surgery RIR is another endourological treatment option for the pelvic ectopic kidneys. Binbay et al. [16] and Bozkurt et al. [17] reported a stone-free rate (SFR) of 70.8% and 84.7% respectively after a single session of RIRS and with no major complications.

Robotic surgery for the ectopic kidneys is less commonly performed for kidney stone management. The use of such a technique showed promising results in reducing postoperative pain, perioperative morbidity, and early return to work [18]. Britt et al. reported the first nephrectomy to be performed using robotic techniques in patients with SIT and the third to use a minimally-invasive approach, with equivalent outcomes to conventional surgical methods [19].

The use of open surgery to treat pelvic kidney stones is high in developing countries such as Pakistan and Iran, in which the rate of pyelolithotomy is as high as 30% in pediatric patients [20, 21]. In the UK the incidence of open renal stone surgery is less than 1% [20].

In developing countries, the use of open surgery upon the non-invasive or minimally invasive approaches is mostly due to the unavailability of the equipment [14].

In our case, open surgery was performed for our patient due to limited resources (lack of endoscopic equipment, and expert hands). The results were promising and the patient was discharged without any serious complications. Similar cases underwent open surgery for EKS and no major complications were observed during and after the procedure [14]. Indeed laparoscopic surgery is a safe and useful method for EKS however in the case of SIT the surgeons might find it difficult to maintain the anatomical orientation [22]. In the literature, few articles that documented the laparoscopic approach in patients with SIT were published, describing a complete laparoscopic kidney removal of a renal mass in a patient with SIT [23]. Additionally, Makiyama and colleagues reported a case of retroperitoneal nephroureterectomy of a patient with SIT using a patient-specific simulator before surgery [24]. Indeed, laparoscopic nephrectomy may be a challenging approach in patients with SIT due to the difficulty in maintaining spatial anatomical orientation. This procedure is more convenient in transperitoneal laparoscopic nephrectomy compared with the retroperitoneal approach, because of the lack of landmarks such as organs in the narrow visible field [24].

Careful preoperative management and rigorous planning are required due to the association between SIT and cardiac, pulmonary, and renal anomalies [25].

## Conclusions

SIT and ectopic kidney associations are rare, and the correlation between these 2 conditions is not well known. The ectopic kidney presents a special diagnostic and therapeutic challenge in this specific situation. Endoscopic approaches might be difficult if accompanied by the SIT.

## Data Availability

Data sharing is not applicable to this article as no datasets were generated or analyzed during the current study. All data (of the patient) generated during this study are included in this published article and its supplementary information files.

## Abbreviations

SIT: Situs inversus totalis
EKS: Ectopic kidney stone
EGU: European guidelines of urology
SWL: Shock wave lithotripsy
PCNL: Percutaneous nephrolithotomy
